# Proteomic and Physicochemical Analysis of Erythrocyte Membrane Vesicles from Fresh and Frozen Blood

**DOI:** 10.1021/acs.jproteome.6c00535

**Published:** 2026-09-09

**Authors:** Katarina Tomić, Sara Ivanovski, Jernej Šribar, Daša Čebulj, Samuel Žvanut, Metka Lenassi, Marko Fonović, Igor Križaj, Nina Kostevšek

**Affiliations:** † Department of Nanostructured Materials, K7, Jožef Stefan Institute, Ljubljana 1000, Slovenia; ‡ 61790 Jožef Stefan International Postgraduate School, Jožef Stefan Institute, Ljubljana 1000, Slovenia; § Department of Biochemistry and Molecular and Structural Biology, B1, Jožef Stefan Institute, Ljubljana 1000, Slovenia; ∥ Department of Molecular and Biomedical Sciences, B2, Jožef Stefan Institute, Ljubljana 1000, Slovenia; ⊥ Faculty of Chemistry and Chemical Technology, University of Ljubljana, Ljubljana 1000, Slovenia; # Institute of Biochemistry and Molecular Genetics, Faculty of Medicine, University of Ljubljana, Ljubljana 1000, Slovenia

**Keywords:** erythrocyte membrane vesicles, blood storage, proteomics, electron microscopy, nano-flow cytometry

## Abstract

Erythrocyte membrane vesicles (EMVs) have emerged as promising nanocarriers for drug delivery due to their biocompatibility and immune evasion through retention of erythrocyte surface proteins. The main limitation is their dependence on freshly drawn blood, which limits batch production. Long-term storage of EDTA-anticoagulated blood at −80°C without cryoprotectant represents an accessible alternative, but its effect on EMV physicochemical properties and protein composition has not been investigated. We prepared EMVs from fresh and frozen blood and characterized the resulting fractions by dynamic light scattering, nano-flow cytometry, cryo-transmission electron microscopy, negative staining transmission electron microscopy, and quantitative mass spectrometry-based proteomics. EMV size, surface charge, and morphology did not differ significantly between fresh and frozen preparations. Extrusion effectively normalized particle size despite increased heterogeneity in frozen ghost cell fractions, and both preparations yielded structurally intact vesicles after ultracentrifugation. At the proteomic level, freezing reduced proteomic complexity in membrane fractions and selectively depleted cytosol-associated proteins. The core membrane proteome, including Band 3, remained stable. However, glycophorin A showed the largest reduction in frozen preparations, and CD47 levels were reduced in the ultracentrifuged fraction. These differences may be critical for applications where preservation of specific surface proteins is required, such as immune evasion or targeted drug delivery. In some cases, frozen blood is potentially a viable source for EMV production when immediate processing is not feasible.

## Graphical abstract

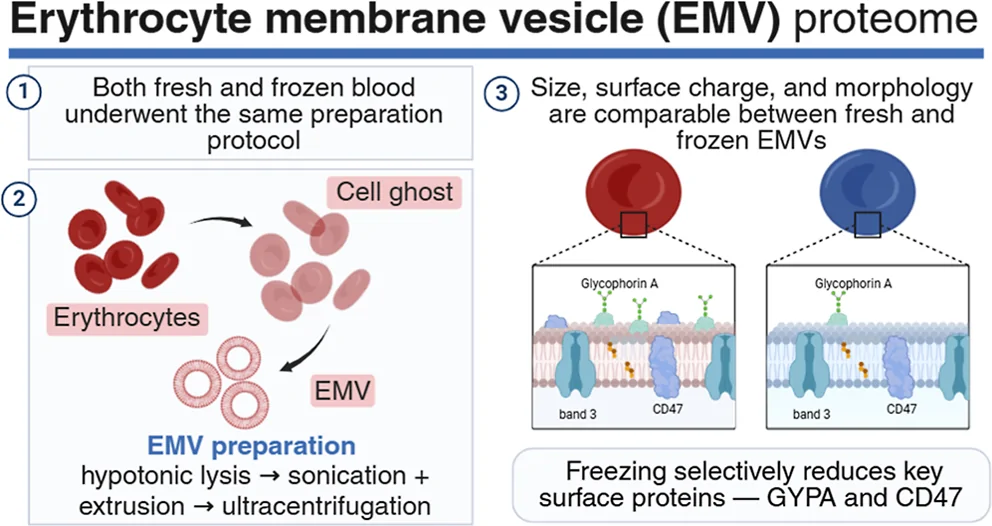

## 1 Introduction

Traditional drug delivery methods are often limited by non-specific distribution and off-target effects in healthy tissues.^1,2^ Nanocarriers like liposomes, inorganic particles, and polymeric micelles improve the solubility of the drug, provide controlled release, and protect the cargo from enzymatic degradation,^1^ but nondegradable materials can accumulate in organs and cause long-term toxicity. Nanoparticles are more likely to be opsonized and rapidly cleared from the systemic circulation by the mononuclear phagocyte system. Surface modifications with polyethylene glycol (PEG) improve circulation but do not mimic the properties of the cell membrane.^3^ These limitations have led to the development of biomimetic delivery systems that mimic the cell membrane to achieve immune evasion and prolonged circulation.^2^

Extracellular vesicles (EVs) are small, lipid-bilayer-enclosed nanoparticles, naturally secreted by all cell types, ranging from 30 to 1000 nm, and are present in all body fluids.^2^ EVs have gained interest as potential delivery systems because of their biocompatibility, low immunogenicity, and ability to cross biological barriers.^2,4^ EVs express surface proteins involved in cell recognition, adhesion, and uptake, allowing targeted delivery without chemical modification.^4^ Naturally shed EVs are heterogeneous, have low and inconsistent yields, and lack standardized isolation protocols, limiting clinical translation.^5,6^ This has driven interest in engineered EV-based systems with improved reproducibility and higher yield.^6^

Among the potential EV sources, red blood cells (RBCs) stand out as promising candidates for clinical translation. RBCs are the most abundant cells in the human body; they lack both mitochondrial and nuclear DNA, reducing the risk of horizontal gene transfer, and possess a spectrin–actin cytoskeleton, giving them deformability.^4^ RBCs express CD47, which inhibits phagocytic clearance by binding SIRPα on macrophages and dendritic cells,^7^ and carry a sialic acid-rich glycocalyx, providing a strong negative surface charge that suppresses protein adsorption and opsonization.^8^ Throughout their lifespan, RBCs naturally shed membrane-derived vesicles, retaining characteristic surface proteins of the parent cell,^9^ making them well-suited for systemic drug delivery requiring prolonged circulation and low immunogenicity.^10^ An autologous blood represents a promising approach for individualized nanomedicine with a reduced risk of immune rejection.^6,9^

To overcome the yield and standardization limitations of naturally shed erythrocyte-derived EVs, engineered erythrocyte membrane vesicles (EMVs), also referred to in the literature as nanoerythrosomes, are produced by hypotonic lysis to deplete intracellular hemoglobin, followed by mechanical fragmentation and sequential extrusion through polycarbonate membranes of defined pore sizes,^6,11^ yielding reproducible vesicle populations, typically in the range of 100–300 nm.^6,10^ This way, the native lipid bilayer composition is preserved, as are some membrane-associated proteins, such as the structural anion transporter Band 3, immune regulatory markers such as CD47, and complement regulatory proteins such as CD55 and CD59 ^12^, as schematically shown in Figure 1. The RBC membrane’s composition is complex, and further research is warranted to gain better insight into potential compositional changes during EMV preparation.

**Figure 1:**
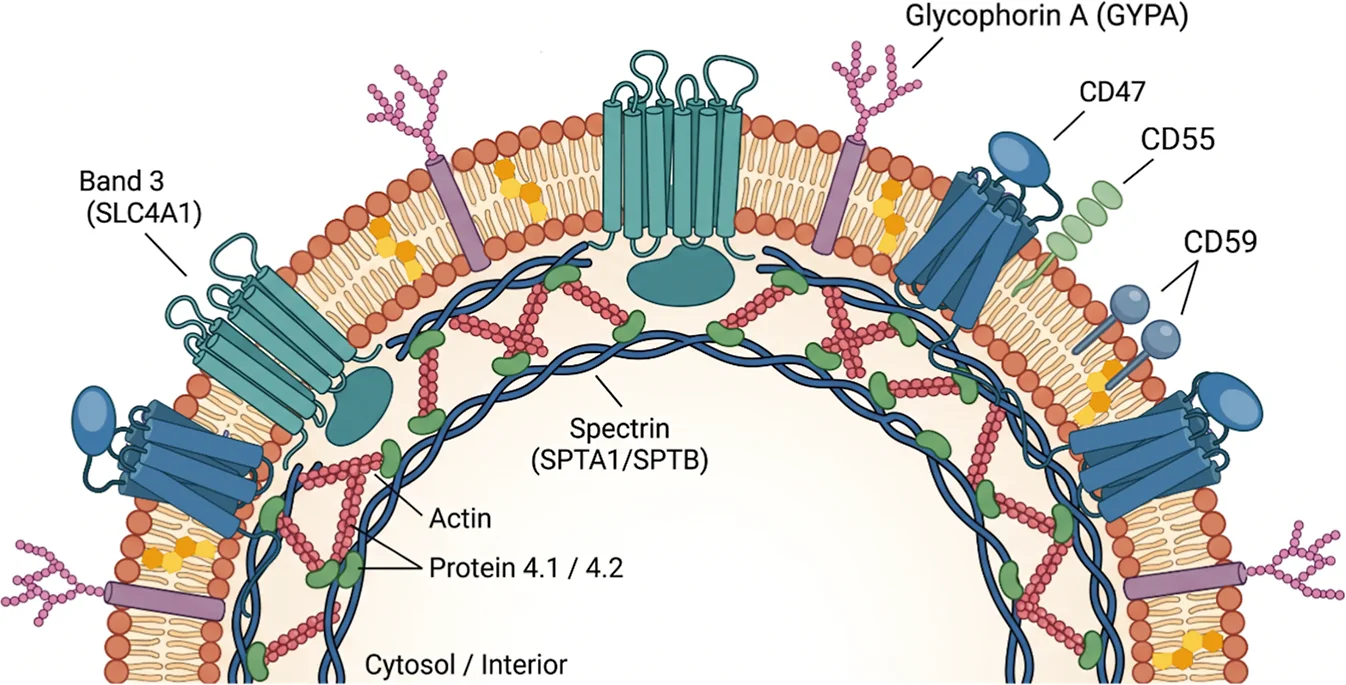
Schematic cross-section of an EMV showing selected key structural and functional membrane proteins. Band 3 is the dominant transmembrane scaffold, glycophorin A carries branching glycan chains on the outer leaflet, CD47 mediates immune evasion, CD55 and CD59 are regulatory proteins, while the inner surface is lined by a spectrin network anchored via actin and proteins 4.1/4.2. Created in BioRender. Tomić, K. (2026) https://BioRender.com/efbstfe

A practical consideration for clinical scalability is the source and storage conditions of the blood used for preparation. Most published studies have used blood bank-stored RBCs (4 °C) or freshly drawn blood as starting material.^11,13–16^ However, the influence of long-term frozen storage on EMV composition and morphology has not been investigated. The aim of our study is to evaluate the physicochemical properties of EMVs, protein profile, and potential protein losses during EMV preparation and purification and to compare profiles across all preparation stages between (a) fresh and (b) −80 °C frozen blood. To the best of our knowledge, this is the first such systematic study to determine whether EMVs made from frozen blood are of equal quality to their fresh counterparts in terms of protein content, morphology, size, and surface charge. The outcome of the study is crucial for future directions in the preparation of EMVs for drug delivery. While the proteomic analyses of whole RBCs have been reported,^17,18^ the proteomics analysis of RBC-derived EVs is scarce. Oh et al. investigated protein profiles between large and small EVs released from 45 day-old RBCs, stored at 4°C.^19^ In addition, Paolini et al.^20^ investigated how long-term storage of isolated EVs influences the size distribution profile and cellular uptake. There is only one proteomic study of EMVs;^21^ even that was on vesicles made of fresh blood. Our study is therefore unique in performing proteomic analysis across all preparation stages, whole erythrocytes (WEs), hemoglobin-depleted cells, EMVs, and ultracentrifuge-purified EMVs, for both fresh and frozen blood. Physicochemical characterization was performed by dynamic light scattering (DLS), nano-flow cytometry, and TEM, including cryo-TEM and negative-stain TEM.

## 2 Materials and Methods

### 2.1 Preparation of EMVs

RBCs were obtained from the Blood Transfusion Center of Slovenia (Ethical approval number: 0120-224/2025-2711-6; informed consent obtained from the donor prior to blood collection) from a young female donor with RhD + blood type, supplemented with CPD-SAGM preservative, 70% v/v hematocrit. EMVs were prepared using two distinct sources of RBCs: freshly isolated erythrocytes and erythrocytes from a single batch of blood stored at −80°C without cryoprotectant for 21 days. Non-processed erythrocytes are named in this study as WEs. Hemoglobin was depleted from erythrocytes using the hypotonic lysis method. The cells were added to 50 mL of 0.1×PBS at a ratio of 0.5 mL of 70% v/v erythrocytes and incubated for 30 min at 4°C to allow hemoglobin release. The suspension was centrifuged at 13,000 rpm using a centrifuge for 7 min at 4°C, and the pellet was washed 4 times under the same conditions to remove residual hemoglobin. This stage sample is named RBC ghosts (GH). GH were finally resuspended in 1 mL of isotonic PBS, sonicated for 10 s (pulses 1 s on, 1 s off; 20% amplitude), and extruded with an Avanti Polar mini extruder through an 800 nm membrane 10 times, followed by a 200 nm membrane 5 times. The extruded sample is called EMVs.

To obtain a high concentration of EMVs while separating EMVs from protein aggregates and cell debris, we performed density-changing centrifugation (DCC) in a Beckman Coulter Optima XPN-90 ultracentrifuge. Ultracentrifuge tubes were filled with 1.5 mL of 10% (m/v) trisodium citrate (TC) dissolved in PBS, onto which 1 mL of EMV sample was gently pipetted to avoid disrupting the two layers. The samples were centrifuged with an SW 60 Ti swinging bucket rotor for 40 min at 4°C at 27,200 rpm. Less dense EMVs remain above the TC cushion, while denser aggregates and cell debris pellet through it. From the third to the sixth of eight 0.25 mL fractions were collected for further analysis. Hereinafter, ultracentrifuged samples are referred to as UC. All stages of sample preparation are schematically shown in Figure 2.

**Figure 2:**
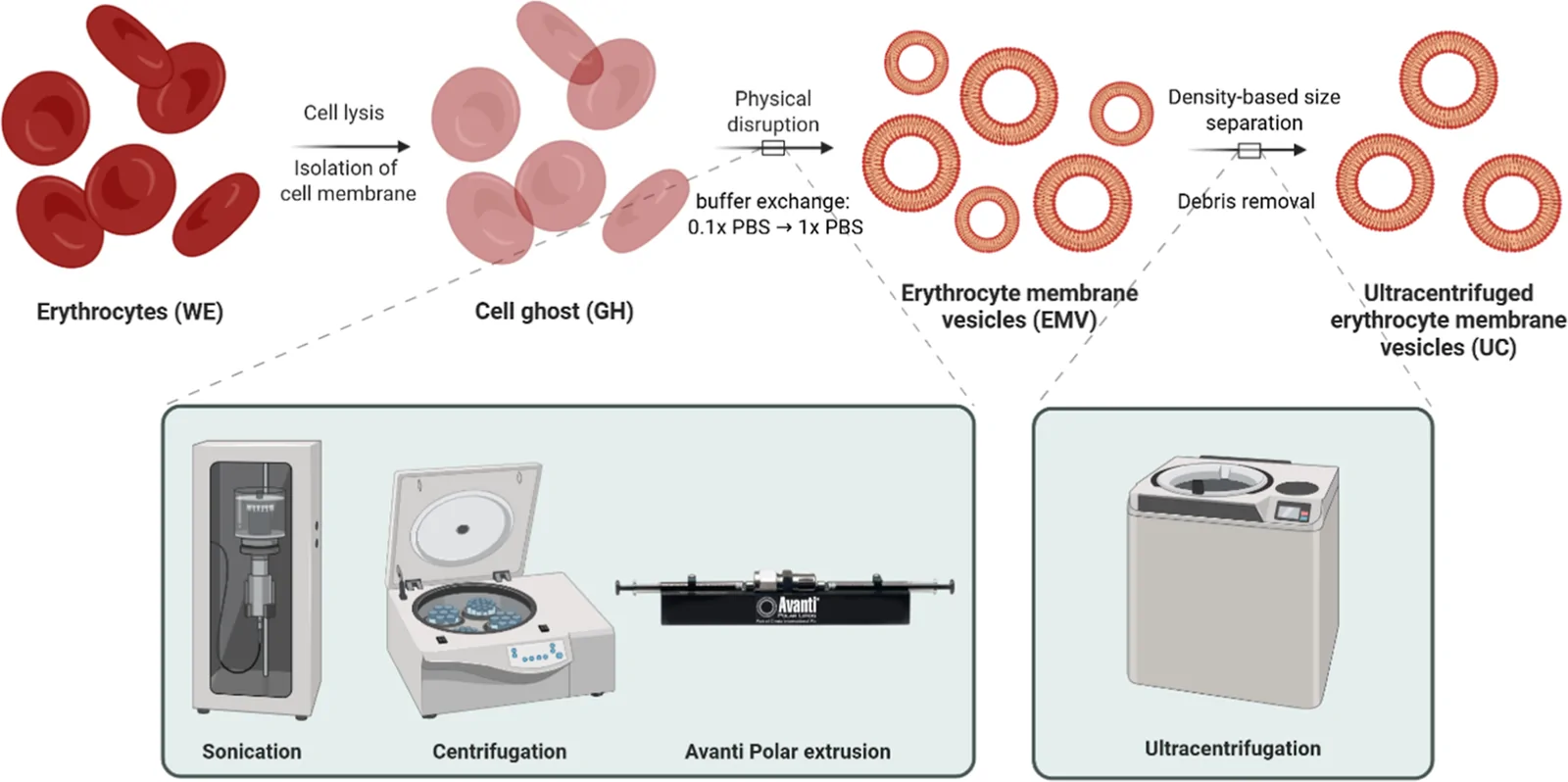
Schematic representation of the erythrocyte membrane vesicle (EMV) preparation protocol applied identically to both fresh and frozen blood. Whole erythrocytes (WE) undergo hypotonic lysis and centrifugation to yield ghost cells (GH), followed by sonication and extrusion through polycarbonate membranes to produce EMVs, and further purification by ultracentrifugation to obtain the UC fraction. Created in BioRender. Tomić, K. (2026) https://BioRender.com/xv3ew5c

### 2.2 Protein Quantification

The bicinchoninic acid (BCA) assay was used according to the supplier’s instructions. As a standard, BSA (Sigma-Aldrich) was used at a stock concentration of 1 mg/mL. The assay-to-sample ratio was 10:1 (v/v), and the 96-well plate was incubated for 2 h at 37°C. All measurements were done in triplicate. The absorbance at 562 nm was measured on transparent 96-well plates on a plate reader, and a calibration curve was generated according to the manufacturer’s guidelines.

### 2.3 Particle Size and Zeta Potential Determination

Zeta potential, hydrodynamic diameter, and polydispersity index (PDI) of GH, EMV, and UC prepared from fresh and frozen blood were determined using a Litesizer DLS 500 particle size and zeta potential analyzer (Anton Paar). The particle size analyzer is equipped with a built-in volume-weight algorithm in proprietary software (Kalliope). Anton Paar Omega cuvettes were used for all measurements. Samples were equilibrated for 10 min and measured in triplicate at 25°C.

### 2.4 Nano-Flow Cytometry

Nano-flow cytometry was performed using the Flow NanoAnalyzer U30E (NanoFCM) to determine particle concentration and size distribution of total particles (EMVs and UC from fresh and frozen blood) and MemGlow640-positive particles according to the manufacturer’s instructions (MemGlow640 membrane dye, Cytoskeleton Inc.). Individual particles were simultaneously detected using single-photon counting modules measuring side scatter (SSC) through a 488/10 nm bandpass filter and red fluorescence through a 670/30 nm bandpass filter. The instrument was calibrated for particle concentration using 250 nm fluorescent silica Quality Control (QC) beads and for particle size using a premixed silica bead standard containing particles of 68, 91, 113, and 155 nm diameter (S16M-Exo beads). EMV and UC samples were diluted 200–2000-fold in Tris–EDTA buffer (pH 7.5) to obtain particle counts within the optimal measurement range (2000–5000 events) during a 1 min acquisition at a pressure of 1 kPa. Data acquisition and particle concentration determination were performed using NanoFCM Professional software v3.0 (NanoFCM). Data were subsequently analyzed using FlowJo v10.10.1 (BD Life Sciences). The percentage of fluorescent particles and particle size distribution were determined, with size distribution reported as mean and median particle diameter.

### 2.5 Ultrastructure Characterization of EMVs

#### 2.5.1 CryoTEM

The EMV and UC samples for cryo-TEM were prepared using a Vitrobot Mark IV (Thermo Fisher Scientific). Quantifoil® R 2/2, 200 mesh holey carbon grids (Quantifoil Micro Tools GmbH) were glow-discharged for 60 s at 20 mA with positive polarity in an air atmosphere (GloQube® Plus, Quorum). The Vitrobot settings were adjusted to 4°C and 95% relative humidity, with a blot time of 6 s and a blot force of 2. Three microliters of the suspension were applied to the grid, blotted, and then vitrified in liquid ethane. The samples were visualized using a 200 kV Glacios microscope (Thermo Fisher Scientific). Micrographs were analyzed for size distribution using ImageJ software and GraphPad Prism 6.01, with size analysis performed at 20,000× magnification.

#### 2.5.2 Negative Staining Transmission Electron Microscopy

EMV and UC samples were prepared for negative staining transmission electron microscopy by mixing with a 1:1 solution of 2.5% glutaraldehyde in cacodylate buffer (0.008 M, pH 7.3) and incubating for 30 min. Subsequently, 5 μL of the suspension was applied to Formvar-coated copper grids (150 mesh, TEMFCF150CU50) for 10 min, after which any excess liquid was gently blotted off using Whatman filter paper. The grids were briefly rinsed by placing them on a water droplet and removing the excess liquid. After that, they were placed on a drop of UA-zero negative stain (Alfa Aesar) for 45 s and left to air-dry. The prepared sample grids were then examined using a JEOL JEM-2100 transmission electron microscope at 200 kV, with images captured at magnifications ranging from 20,000× to 40,000x.

### 2.6 Mass Spectrometry-Based Proteomics

WE, GH, EMV, and UC fractions from fresh and frozen blood samples were prepared according to an in-gel digestion protocol. All samples were measured in technical triplicates. Briefly, samples were denatured at 95°C in a 6x loading buffer and then separated on an 8–16% precast SDS-PAGE gel (Invitrogen). A gel grid cutter was used to cut each sample lane into 30 uniformly sized bands. Two bands corresponding to hemoglobin were removed, and the rest were combined into 4 fractions per sample, each containing seven gel bands. The following preparation steps of in-gel digestion and peptide recovery were performed as specified previously by Kolarič et al.^22^

#### 2.6.1 Mass Spectrometry Analysis

Peptides were separated by nanoliquid chromatography (Thermo Scientific Vanquish Neo) coupled with a mass spectrometer (Thermo Scientific Exploris 480) and with an electrospray source (Thermo Scientific NanoSpray Flex NG ion source). Peptides were trapped onto a precolumn (Thermo Scientific μPAC Trapping Column) using solvent A (0.1% formic acid, MS grade). Then, peptides were separated on an analytical column (Thermo Scientific μPAC NEO HPLC Column, 50 cm NanoLC) using a 75 min linear gradient from 0% to 45% of solvent B (0.1% formic acid in 80% acetonitrile, MS grade) at a flow rate of 0.5 μL/min. Raw data were acquired on a mass spectrometer in data-dependent acquisition mode. Full-scan MS spectra were acquired on the Orbitrap (scan range, 300–1800 *m*/*z*; resolution, 90,000; automatic gain control target, 3 × 106; and maximum injection time of 25 ms). High-intensity peaks were selected for higher-energy collision dissociation (HCD), with a total cycle time of 1.5 s at 30% normalized collision energy. HCD spectra were acquired on the Orbitrap at a resolution of 15,000, with an AGC target of 2 × 105 and a maximum injection time set to automatic. Charge inclusion was selected for ions with charge states of 2–6. The dynamic exclusion was set to automatic.

#### 2.6.2 Mass Data Processing and Analysis

Tandem mass spectra were analyzed in MaxQuant with an embedded Andromeda search engine.^23^ The search was performed against the UniProt *Homo sapiens* reviewed database containing 20406 entries (downloaded in December 2024). Trypsin-specific cleavage sites were searched with a maximum of two missed cleavages. The search engine parameters considered carbamidomethylation of cysteine residues (+57.0215) as a fixed modification and methionine oxidation (+15.9949) and N-terminal acetylation (+42.0106) as variable modifications. The peptide mass tolerance was set to 20 ppm during the first search and 4.5 ppm during the main search. For protein identification, the false discovery rate was set to 1%. Quantification was determined by using label-free quantification with default settings.

The obtained results were processed in Perseus (version 2.1.3.0).^24^ First, reverse decoy matches, potential contaminants, proteins identified only by site, and proteins with >1 valid value per sample type were filtered out. The resulting expression matrix, containing 751 proteins, was log2-normalized. Correlation analyses were assessed using Pearson’s correlation coefficient (r), and Venn diagrams were prepared for each pair of fresh–frozen sample types. An enrichment analysis tool from the PANTHER classification system through the Gene Ontology (GO) website was used to identify enriched terms.^25^ Graphs were prepared in GraphPad Prism version 10.6.1.

### 2.7 Statistical Analysis

Statistical analyses were performed using GraphPad Prism (version 6.01). Normality of data distribution was assessed using the D’Agostino–Pearson omnibus test. For particle size and zeta potential measurements obtained by DLS, group comparisons between fresh and frozen preparations were performed using an unpaired *t*-test with Welch’s correction. For particle size measurements obtained from cryo-transmission electron microscopy and negative staining transmission electron microscopy micrographs, data did not follow a normal distribution, and group comparisons were therefore performed using the Mann–Whitney *U* test. Results are expressed as mean ± standard deviation (SD) for normally distributed data and as median with interquartile range (IQR) for non-normally distributed data. A *p*-value <0.05 was considered statistically significant. Figures were prepared using OriginPro (version 2021).

### 2.8 Image Analysis and Size Measurements

Particle sizes were determined by manual measurement from transmission electron microscopy micrographs using ImageJ software (version 1.54g). For cryo-TEM size analysis, measurements were performed on at least six micrographs acquired at 20,000× magnification, with a total of 70 particles measured per sample group. For negative staining TEM size analysis, measurements were performed on micrographs acquired at magnifications between 20,000× and 40,000×, with a total of 170 particles measured per sample group. The length of each particle was measured using the straight-line tool in ImageJ, with the scale bar used for calibration.

## 3 Results

### 3.1 Extrusion Normalizes Particle Size Regardless of Blood Storage Condition

To evaluate the impact of mechanical processing on particle size and homogeneity, DLS was performed on all sample types (Figure 3).

**Figure 3:**
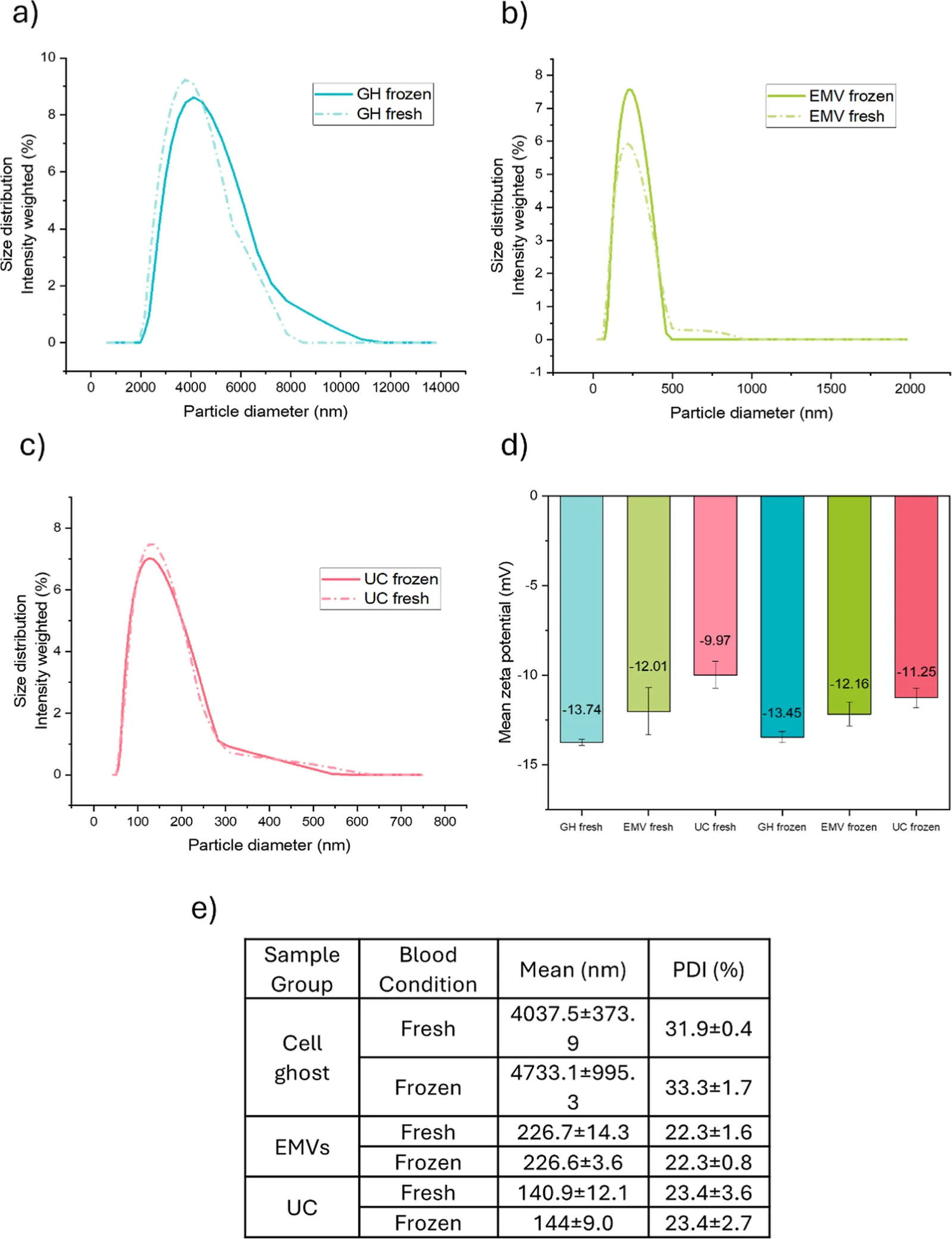
Particle size characterization of GH, EMV, and UC fractions by DLS. Size distributions for fresh (dashed) and frozen (solid) preparations (a–c), mean zeta potential (d), and summary of mean hydrodynamic diameter and PDI for all sample groups (e); data presented as mean ± SD (*n* = 3).

Fresh GH showed a mean hydrodynamic diameter of 4037 ± 374 nm with a PDI of 31.9 ± 0.4%, consistent with the large, irregularly shaped membrane sheets expected following hypotonic lysis. GH prepared from frozen blood showed a comparable mean diameter of 4733 ± 995 nm with a PDI of 33.3 ± 1.7% (Figure 3a). No statistically significant difference was observed between fresh and frozen GH for either parameter (unpaired *t*-test with Welch’s correction, *p* > 0.05). The higher SD in the frozen group reflects greater batch-to-batch variability, consistent with Ahmadian et al., who reported that the freeze-thaw cycle induces membrane disruption, ice crystal formation, and osmotic stress during freezing.^26^ Following sonication and sequential extrusion through 800 and 200 nm polycarbonate membranes, both fresh and frozen preparations yielded EMVs with nearly identical mean hydrodynamic diameters of 226.7 ± 14.3 nm and 226.6 ± 3.6 nm, with a PDI of 22.3 ± 1.6% (fresh).

This demonstrates that extrusion through polycarbonate membranes reduces erythrocytes of ∼7.5–8.7 μm diameter^6^ and ghost cells of several micrometers to vesicles of 100–200 nm by mechanical fragmentation and membrane reorganization, consistent with extrusion-based EMV production reported by others.^8,11,27^ After density-gradient ultracentrifugation, the mean diameter decreased further to 140.9 ± 12.1 nm (fresh) and 144.0 ± 9.0 nm (frozen), with PDI values of 23.4 ± 3.6% and 23.9 ± 2.7%, respectively (Figure 3c), reflecting isolation of a more homogeneous vesicle subpopulation. The ∼3 nm difference in mean diameter between fresh and frozen UC fractions, though statistically significant (*p* < 0.05), is not biologically relevant for vesicles in this size range. All measured mean diameters and PDI values are summarized in Figure 3e.

Zeta potential measurements were performed on all sample groups to assess surface charge (Figure 3d). All groups exhibited negative zeta potential values across all preparation steps, with no statistically significant differences between fresh and frozen preparations at any stage (unpaired *t*-test with Welch’s correction, *p* > 0.05 for all comparisons). Fresh and frozen GH showed a mean zeta potential of −13.74 ± 0.18 mV and −13.45 ± 0.31 mV, respectively, comparable to native erythrocytes, where zeta potential is around −15 mV in young cells, and it decreases to approximately −12 mV in senescent cells.^28^

Following extrusion, fresh EMVs showed −12.01 ± 1.31 mV and frozen EMVs −12.16 ± 0.66 mV. After ultracentrifugation, values were −9.97 ± 0.75 mV (UC fresh) and −11.25 ± 0.55 mV (UC frozen). The gradual decrease from GH to UC fractions is possibly due to the removal of charged surface material during ultracentrifugation. The negative surface charge of RBC-derived vesicles is mainly due to sialic acid residues on membrane glycoproteins and glycolipids, while negatively charged phospholipid headgroups contribute additionally. This surface charge reduces non-specific interactions with plasma proteins, modulating protein corona composition and prolonging circulation time.^29,30^ The values across all stages are within the ranges reported for nanosized erythrocyte ghosts^21^ and purified EMVs,^10,11^ indicating that both preparations maintain sufficient surface charge for particle stability in physiological buffers.

To complement DLS measurements and obtain particle-resolved size and concentration data, nano-flow cytometry was performed on EMV and UC fractions using MemGlow640 membrane dye to identify particles with a lipid bilayer (Figure 4). In heterogeneous samples, DLS is biased toward larger particles, which dominate the scattered light signal and obscure the smaller population,^6^ while nano-flow cytometry detects individual particles down to ∼40 nm, providing a number-weighted size distribution that accurately represents the most abundant population.^31^

**Figure 4:**
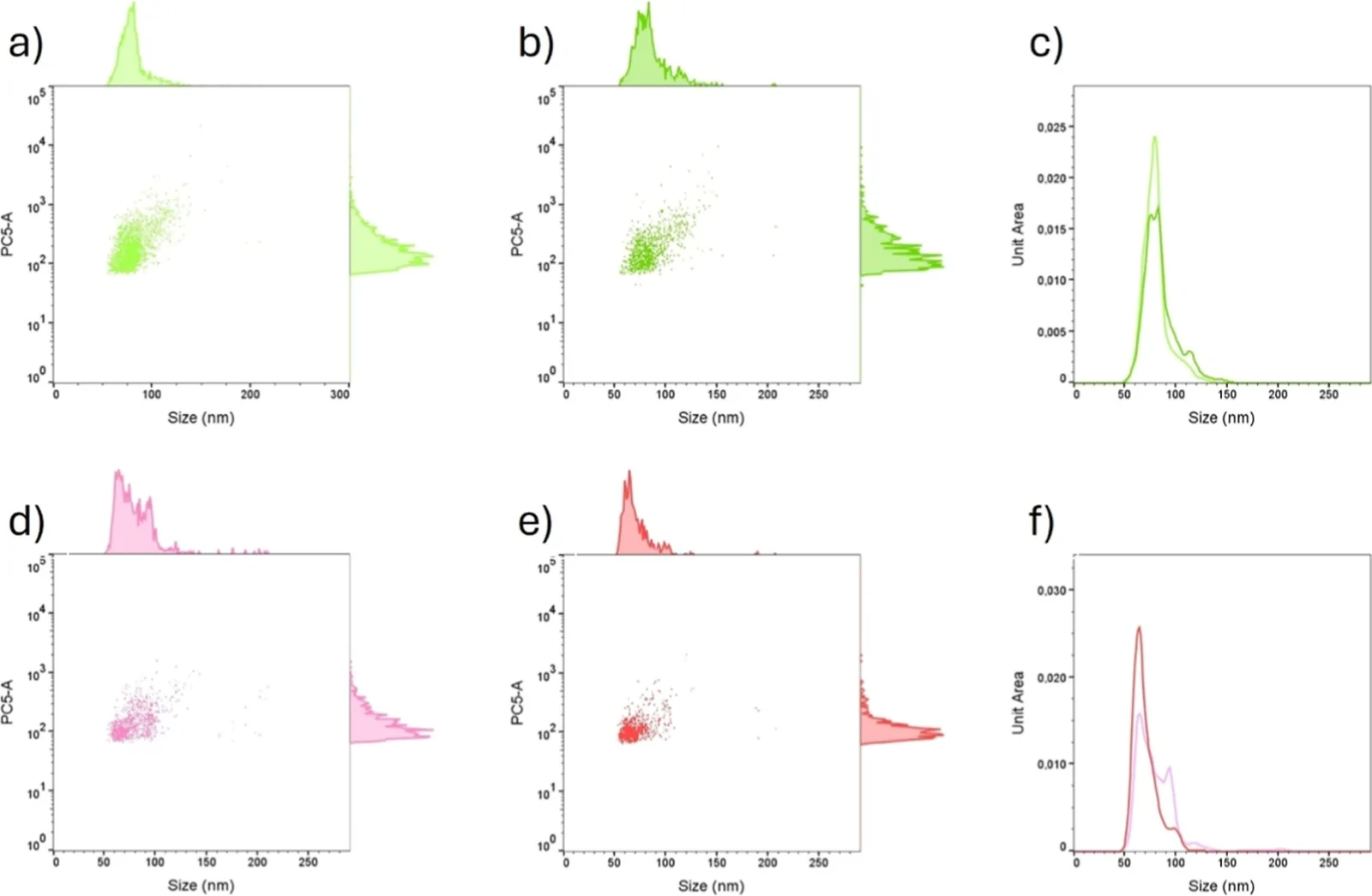
Nano-flow cytometry analysis of MemGlow640-positive particles from EMV and UC fractions derived from fresh and frozen blood. Scatter plots (a,b,d,e) show particle size versus PC5-A fluorescence intensity; size distribution histograms compare fresh and frozen MemGlow640-positive particles for EMV (c) and UC (f) fractions, with *y*-axes normalized to unit area.

Nano-flow cytometry analysis of EMV fractions revealed median particle diameters of 74.8 nm (fresh) and 73.8 nm (frozen), with mean diameters of 75.5 ± 10.5 nm and 75.3 ± 12.0 nm, respectively, with no statistically significant differences between groups. This is consistent with the smaller vesicle population identified by nano-flow cytometry in RBC-derived preparations.^32^ Nanoparticle tracking analysis (NTA) measurements of similar preparations report sizes in the 100–300 nm range,^33^ which is expected, given that NTA has a higher minimum detectable particle size than nano-flow cytometry and therefore preferentially detects larger particles within heterogeneous nanoparticle populations.

UC fractions showed slightly smaller median diameters of 66.2 nm (fresh) and 64.8 nm (frozen). Particle concentrations were 1.64 × 10^11^ particles/mL (EMV fresh), 2.39 × 10^11^ particles/mL (EMV frozen), 2.96 × 10^10^ particles/mL (UC fresh), and 4.84 × 10^10^ particles/mL (UC frozen). The proportion of MemGlow-positive particles decreased following ultracentrifugation, from 53.1% to 24.1% in fresh and from 34.7% to 27.1% in frozen samples. This suggests that, besides intact membrane vesicles, the starting suspensions likely contained lipid-rich non-vesicular particles, membrane fragments, and membrane aggregates that can also associate with lipophilic dyes and contribute to fluorescent events. Ultracentrifugation under density-gradient conditions recovers only particles within a defined density and sedimentation coefficient window, which is consistent with this selective enrichment and with yield losses documented in other EV purification protocols.^11^ PBS and TE buffer controls showed 0% MemGlow + events, but since these controls did not contain MemGlow, they do not really confirm assay specificity. We also tested the MemGlow control you provided (100-fold dilution in TE buffer). Although 65.6% of the detected particles were MemGlow+, the total particle count was very low (387), comparable to the buffer controls, suggesting that the dye itself does not contribute significantly to the detected signal. Structural integrity of the retained vesicle population was confirmed by cryo-TEM.

### 3.2 Fresh and Frozen EMVs Form Intact Vesicular Structures with Morphological Differences Resolving after Ultracentrifugation

#### 3.2.1 CryoTEM

To evaluate the structural integrity and morphological characteristics of the EMVs and UC fractions, cryo-transmission electron microscopy (cryo-TEM) was performed on both fresh and frozen blood sources (Figure 5). All images were acquired at a magnification of 57,000 x, corresponding to a 100 nm scale bar. Spherical unilamellar vesicles are the dominant morphology in both fresh and frozen EMVs, with diameters ranging from approximately 50 to 300 nm, consistent with DLS measurements and cryo-TEM findings from other RBC-derived vesicle preparations.^27,34^ Multilamellar structures were also observed in fresh EMVs in which smaller vesicles were enclosed within a larger membrane shell. Such structures have been described in EV preparations from other sources^35,36^ and are likely caused by incomplete membrane reformation during extrusion. In both preparations, a subset of vesicles contains an electron-dense luminal material, likely residual hemoglobin that was not fully removed during hypotonic lysis. Consistent with cryo-TEM images of hemoglobin-containing RBC-derived vesicles.^34,37^ Small electron-dense spherical structures without a discernible lipid bilayer were additionally present (red arrows), likely representing nonvesicular material coisolated during preparation.

**Figure 5:**
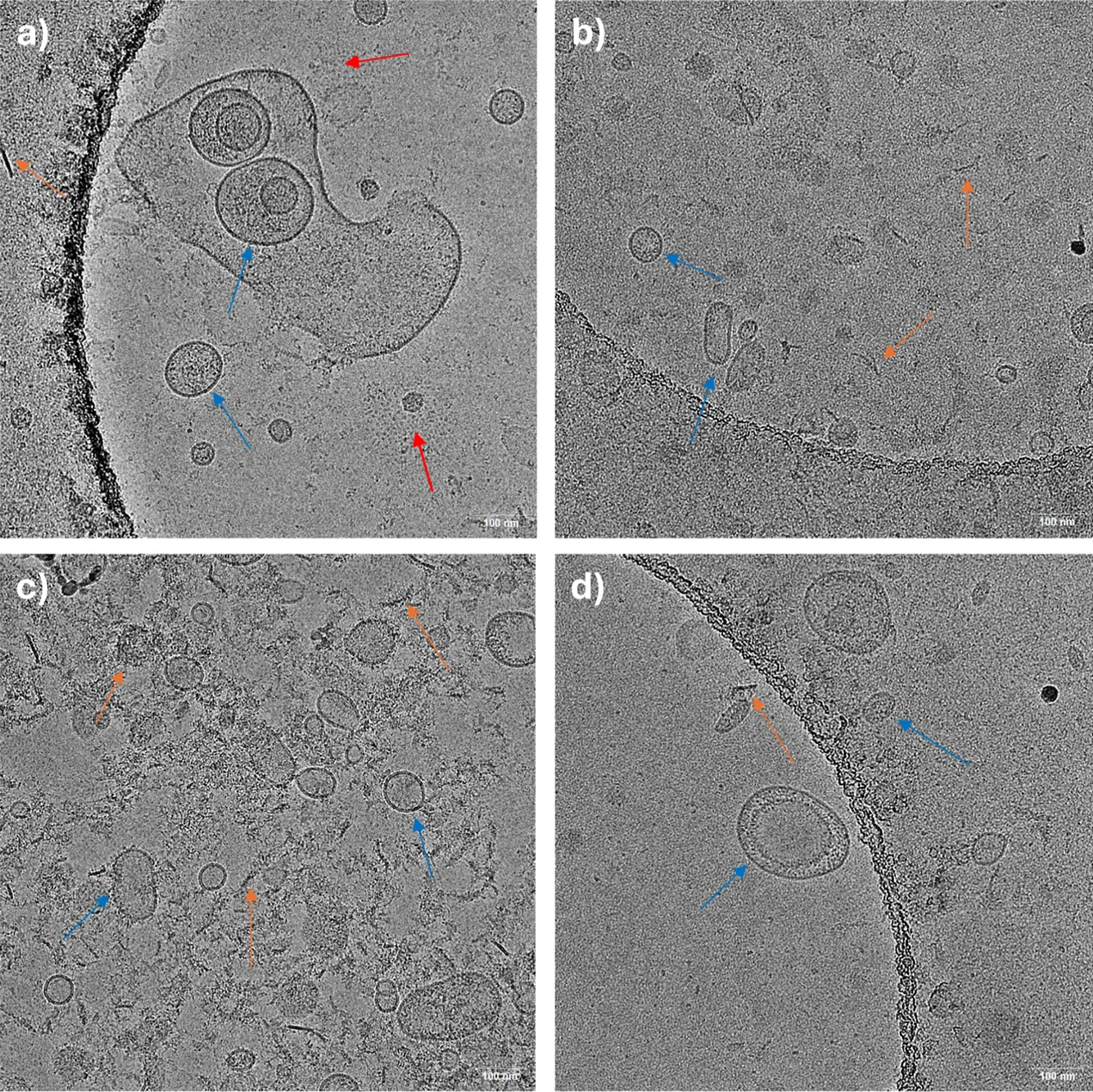
Cryo-TEM micrographs of EMVs and UC fractions from fresh (a,b) and frozen (c,d) blood. Blue arrows: intact vesicles; orange arrows: membrane fragments and debris; red arrows: electron-dense non-vesicular structures. Scale bars: 100 nm.

UC fractions from fresh blood (Figure 5b) exhibited a visibly reduced particle density, consistent with the decrease in total particle concentration measured by nano-flow cytometry. The remaining particles were predominantly spherical and displayed greater size uniformity. In addition to spherical unilamellar vesicles, elongated membrane structures were also observed. The EMVs isolated from frozen blood (Figure 5c) had a higher density of particles per field compared to fresh EMVs, consistent with the increased particle concentration measured by nano-flow cytometry (2.39 × 10^11^ vs 1.64 × 10^11^ particles/mL), and contained a higher density of membrane fragments, irregular structures, and elongated vesicles. This increased morphological heterogeneity can be attributed to the physical damage erythrocyte membranes sustained during freezing without a cryoprotectant, corresponding to the increased size variability observed by DLS in frozen GH, and is in line with freeze-induced damage reported for EVs from other sources.^26^ Despite this, intact bilayer-enclosed vesicles remained clearly identifiable. UC fractions from frozen blood (Figure 5d) showed lower particle density compared to frozen EMVs, consistent with nano-flow cytometry concentration data, with a large unilamellar vesicle with a clearly defined single membrane being prominently observed alongside smaller spherical vesicles. Across all preparations, cryo-TEM confirmed the successful formation of vesicular structures with a membrane bilayer. The ultracentrifugation step reduced membrane debris in both fresh and frozen preparations, producing structurally intact vesicles as the major population. While ultracentrifugation removed most debris to yield intact vesicles in both preparations, the higher debris content in frozen samples may reduce the overall yield of usable EMVs, which could be a relevant consideration for applications such as drug encapsulation.

#### 3.2.2 Negative Staining Conventional Transmission Electron Microscopy

Morphological characterization via TEM confirmed that both fresh and frozen samples consisted of intact, cup-shaped vesicles (Figure 6), a morphology attributable to a dehydration artifact caused by vesicles collapsing during drying.^38,39^ EMVs from fresh blood (Figure 6a) showed well-separated vesicles of varying diameters with clearly defined membrane contours. EMVs from frozen blood (Figure 6b) displayed a similar morphology, with a visibly higher particle density per field of view, consistent with the higher particle concentration observed by nano-flow cytometry. Figures 6c,d shows UC fractions obtained from fresh and frozen blood, respectively, which showed the same cup-shaped morphology. Ultracentrifugation reduced membrane debris in both fresh and frozen preparations, and UC fractions from both storage conditions showed predominantly spherical vesicles. This suggests that ultracentrifugation partially compensates for the morphological differences introduced by blood freezing.

**Figure 6:**
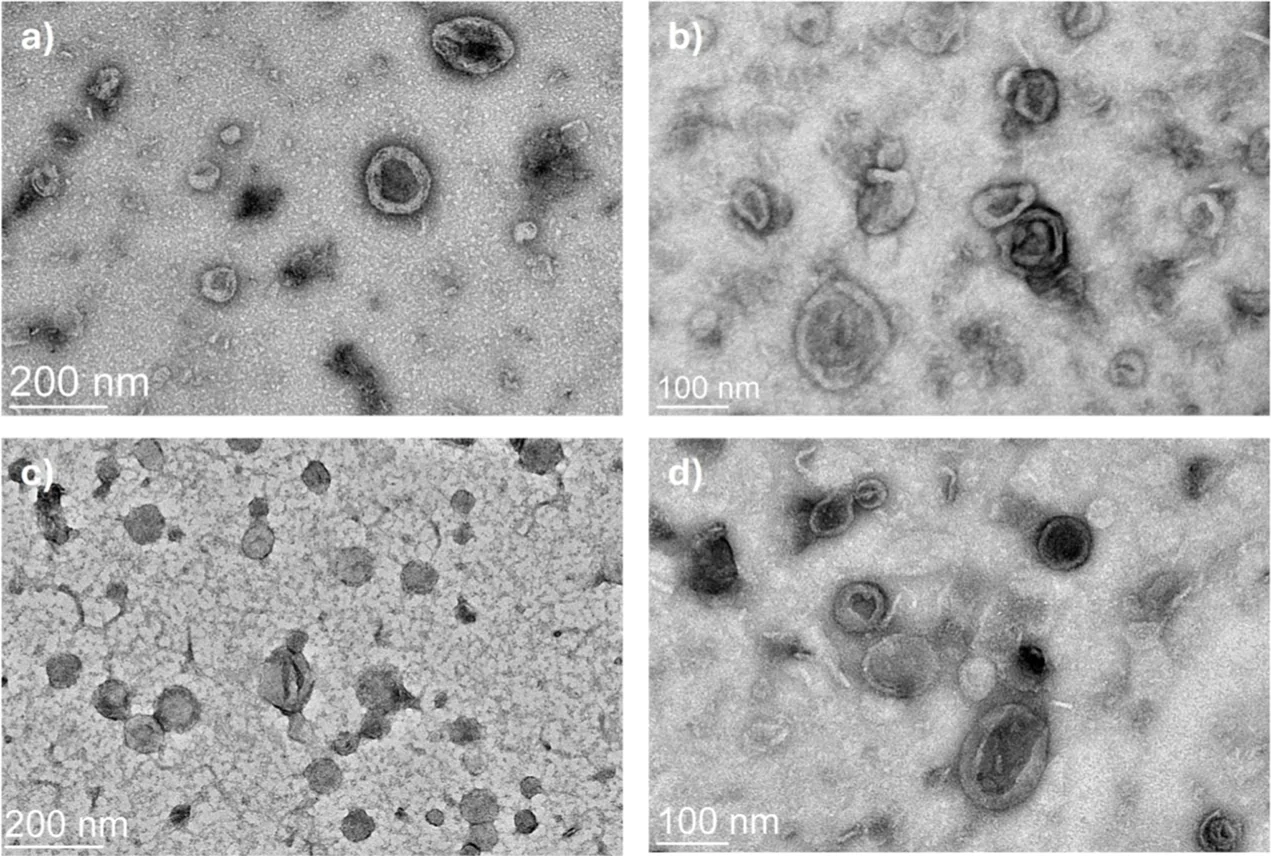
Negative-staining TEM micrographs of EMVs and UC fractions from fresh (a,c) and frozen (b,d) blood showing cup-shaped vesicle morphology. Scale bars: 200 nm (a,c) and 100 nm (b,d).

Particle sizes measured from cryo-TEM micrographs by manual ImageJ analysis are presented in Figure 7a. The median particle size of EMV fresh was 69.33 nm (IQR: 52.55–128.5 nm), and that of EMV frozen was 73.68 nm (IQR: 55.25–95.15 nm), with no statistically significant difference between the two groups (Mann–Whitney *U* test, *p* = 0.9). UC fresh fractions showed a median size of 59.62 nm (IQR: 40.43–89.91 nm), which was significantly smaller than EMV fresh (*p* = 0.0042). UC frozen fractions had a median size of 73.21 nm (IQR: 61.50–112.6 nm), which did not differ significantly from EMV frozen (*p* = 0.4). UC fresh and UC frozen fractions differed significantly from each other (*p* = 0.0012), pointing to an effect of blood storage condition on the vesicle subpopulation selected by ultracentrifugation.

**Figure 7:**
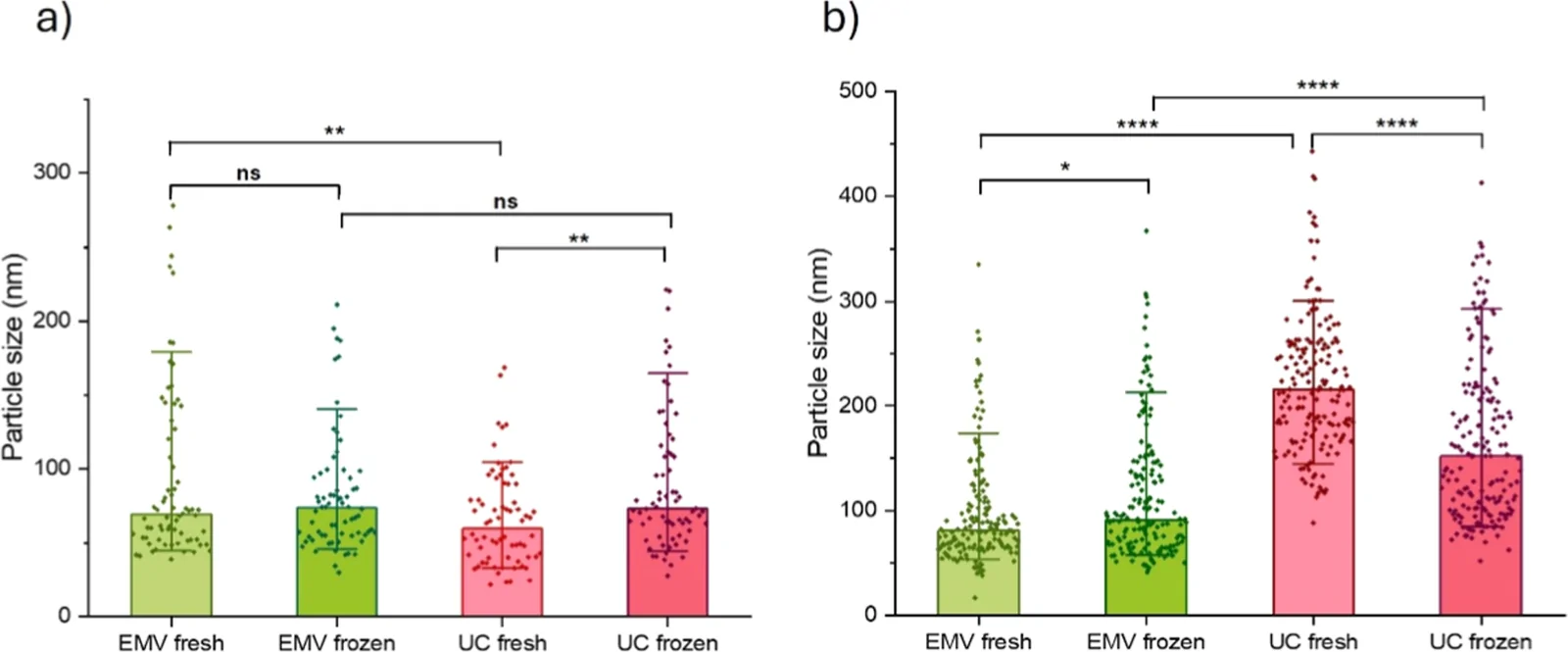
Particle size distributions of EMV and UC fractions measured from (a) cryo-TEM and (b) negative-staining TEM micrographs by manual ImageJ analysis (*n* = 70 and *n* = 170 per group, respectively). Data are shown as median with the 10th–90th percentile range; Mann–Whitney *U* test: *p < 0.05, ****p < 0.0001, ns: not significant.

Particle sizes obtained from negative-stain TEM micrographs are shown in Figure 7b (*n* = 170 per group). Data were non-normally distributed in both groups, as confirmed by the D’Agostino–Pearson omnibus test (*p* < 0.0001 for both groups). The median particle size of EMV fresh was 81 nm (IQR: 65–111 nm), and that of EMV frozen was 91 nm (IQR: 68–141 nm), with a statistically significant difference between the two groups (Mann–Whitney *U* test, *p* = 0.0259), with frozen EMVs showing a larger median diameter and wider size distribution. This difference was absent in cryo-TEM, consistent with cryo-TEM better preserving native morphology and avoiding freeze-thaw artefacts that may exaggerate size differences in NS-TEM. Following ultracentrifugation, UC fresh fractions showed a median size of 216 nm (IQR: 181–260 nm), which was significantly larger than EMV fresh (*p* < 0.0001). UC frozen fractions had a median size of 152 nm (IQR: 102–213 nm), which was significantly larger than EMV frozen (*p* < 0.0001). UC fresh and UC frozen fractions differed significantly from each other (*p* < 0.0001), with UC fresh showing a larger median diameter than UC frozen. These NS-TEM images show significantly larger particle sizes (216 and 152 nm for fresh and frozen UC, respectively), compared to cryo-EM sizes (59 and 73 nm for fresh and frozen UC, respectively). According to MISEV2018 guidelines,^40^ cryo-TEM is considered the gold standard and more reliable method for determining the native size and shape of EVs, as it avoids the dehydration and staining artifacts, as negative staining can cause significant overestimation of diameter due to flattening. In our study, too, it is evident that cryo-TEM sizes correspond well with DLS and nano-flow cytometry data.

### 3.3 Proteomic Analysis

Protein expression profiles in WE, GH, EMV, and UC for fresh and frozen blood preparations are visualized by SDS-PAGE gels in Figure S1 in the Supporting Information file. The corresponding lanes in the gels were used for proteomics analysis.

#### 3.3.1 Protein Groups

The protein composition of samples collected at each stage of the EMV preparation process was analyzed with mass-spectrometry-based proteomics to assess the effect of blood storage on the proteomic profile (Supporting Information Table S1). The number of detected proteins at each preparation stage is shown in Figure 8a.

**Figure 8:**
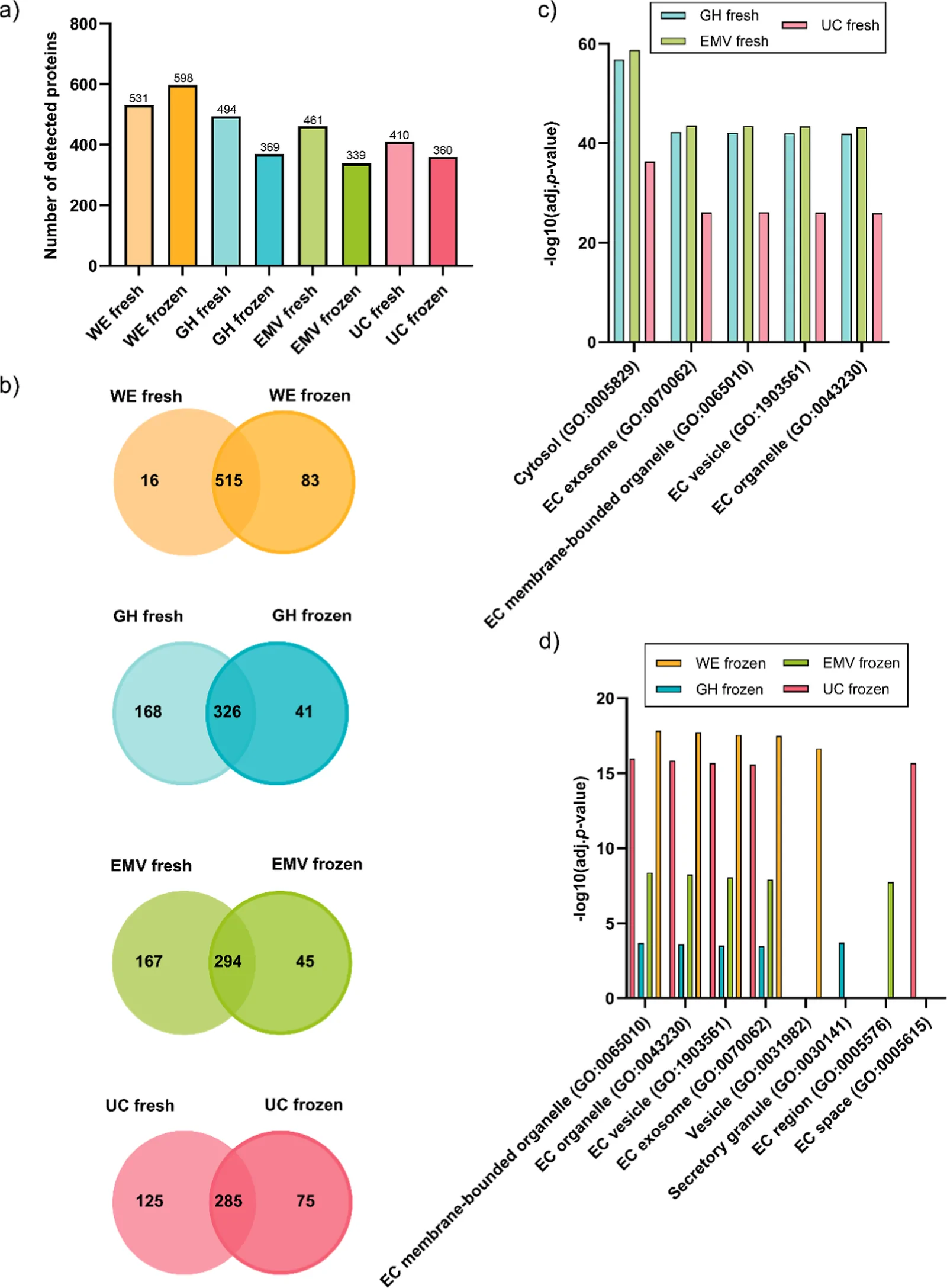
Proteomic analysis across EMV preparation stages. (a) Number of detected proteins per fraction, (b) Venn diagrams of shared and unique proteins between fresh and frozen preparations, and GO enrichment analysis of cellular component terms for proteins uniquely detected in (c) fresh and (d) frozen samples. WE: whole erythrocyte, GH: ghosts, EC: extracellular.

In the fresh blood group, the highest number of proteins was detected from the WE fraction (*N* = 531), while the number of proteins decreased from the GH fraction (*N* = 494) to the EMV (*N* = 461) and finally to the UC fraction (*N* = 410). A similar decreasing trend was observed in frozen blood samples, with the exception of the WE fraction, where the number of detected proteins was higher in frozen than in fresh blood (WE frozen, *n* = 598). For the remaining stages, frozen samples consistently yielded fewer detected proteins than their fresh counterparts: GH (369), EMV (339), and UC (360). This inversion at the WE stage most likely reflects interference of released hemoglobin with the BCA protein assay used to normalize the protein loading. Freezing of erythrocytes without a cryoprotectant induces partial hemolysis, releasing intracellular hemoglobin into the suspension. Hemoglobin is known to interfere with BCA quantification by absorbing at the assay wavelength, potentially causing a systematic overestimation of protein concentration. Consequently, this could result in the erroneous detection of more unique proteins in the frozen WE fractions. The reduction in detected proteins in frozen membrane fractions at the GH, EMV, and UC stages is more biologically informative and suggests loss of proteomic complexity in the membrane compartment, consistent with reports of protein aggregation, oxidative modification, and altered membrane–protein associations during blood storage.^13,41^

Next, a pairwise Venn diagram was used to visualize shared and unique proteins between the fresh and frozen samples (Figure 8b). For each pair, the majority of identified proteins were in the intersection (WE, *N* = 515, GH, *N* = 326, EMV, *N* = 294, UC, *N* = 285). More unique proteins were identified in the fresh samples (GH, *N* = 168, EMV, *N* = 167, UC, *N* = 125) than in the frozen samples (GH, *N* = 41, EMV, *N* = 45, UC, *N* = 75), except for the WE fraction, where more unique proteins were identified in the frozen samples (WE, *N* = 83) than in the fresh samples (WE, *N* = 16).

GO enrichment analysis of the cellular component category was performed on proteins uniquely detected under each condition and revealed two distinct patterns. In fresh blood samples (Figure 8c), the cytosol term (GO:0005829) was enriched exclusively in fresh preparations. Peripherally membrane-associated cytoplasmic proteins that are retained in fresh GH preparations are therefore selectively depleted in frozen preparations. The next pattern represents the terms associated with extracellular cell components. Four terms were enriched in both fresh and frozen samples (Figure 8c,d): EC exosome (GO:0070062), EC membrane-bound organelle (GO:0065010), EC vesicle (GO:1903561), and EC organelle (GO:0043230). This confirms that the detected proteins in both preparations were associated with vesicular structures rather than representing nonvesicular contamination.

#### 3.3.2 Correlation Matrix

To assess the similarity between proteomic profiles at each preparation stage, Pearson correlation coefficients (r) were calculated between WE, GH, EMV, and UC fractions for both fresh and frozen preparations (Figure 9a,b). The highest correlation coefficients were observed between GH and EMV samples in both fresh (*r* = 0.94) and frozen (*r* = 0.95) blood samples. Additionally, a very high coefficient was determined between GH and UC (fresh: *r* = 0.84, frozen: *r* = 0.84) as well as EMV and UC samples (fresh: *r* = 0.89, frozen: *r* = 0.84). These results confirm that hypotonic lysis, extrusion, and ultracentrifugation collectively enrich the pool of membrane-associated proteins.

**Figure 9:**
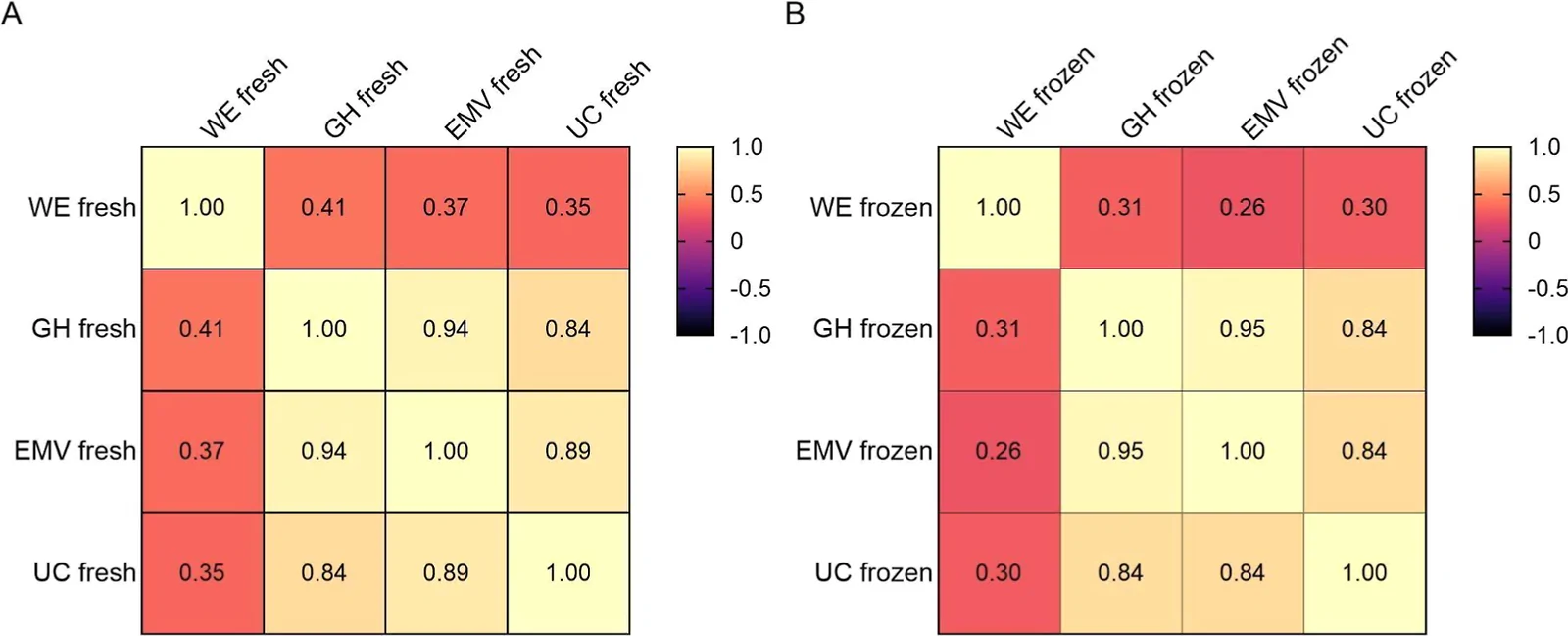
Pearson correlation coefficient (r) matrix of proteomic profiles across preparation stages for (a) fresh and (b) frozen blood samples. Correlation coefficients are shown for WE, GH, EMV, and UC samples. The color scale represents the Pearson correlation coefficient from −1 (dark purple) to 1 (light yellow). WE: whole erythrocyte, GH: ghosts, EMV: erythrocyte membrane vesicles, UC: ultracentrifuged samples.

Conversely, all of the comparisons with WE samples had very low coefficients in both the fresh (GH: *r* = 0.41, EMV: *r* = 0.37, UC: *r* = 0.35) and frozen blood samples (GH: *r* = 0.31, EMV: *r* = 0.26, UC: *r* = 0.30). This low correlation reflects the abundance of hemoglobin in the WE fraction, which constitutes approximately 97% of total RBC protein by mass and disables the detection of membrane-associated proteins, which are several orders of magnitude lower in abundance.^18,41^ Taken together, our results indicate that the proteomic profile of the preparation is established at the GH cell stage and remains largely preserved through subsequent steps, whereas the WE proteomes differ significantly.

The WE proteomes were the most distinct from the membrane-enriched fractions at all preparation stages under both fresh and frozen conditions (Figure 9). Correlation coefficients among GH, EMV, and UC fractions were high or very high under both fresh and frozen conditions.

The present proteomics data are broadly consistent with findings from Böröczky et al., who showed that storage of EV isolates at −80°C had no adverse effect on mass spectrometry-based protein identification in melanoma cell-derived small EVs, with 99.3% overlap between fresh and frozen EV proteomes and a correlation coefficient of 0.95 ^42^. Similarly, D’Arrigo et al. demonstrated that EVs isolated from fresh and frozen human melanoma tissue were similar in protein composition, with no significant enrichment of intracellular contaminants in frozen samples.^43^ However, both of those studies compared the effect of freezing on already-isolated EVs or on tissue as starting material, whereas our study uniquely examines the effect of freezing the blood source itself across all stages of EMV preparation and purification.

#### 3.3.3 Fold Change: Frozen vs Fresh Samples

To evaluate the effect of blood storage on the abundance of key erythrocyte membrane proteins, log2 fold changes (frozen relative to fresh) were calculated for a selected panel of proteins across GH, EMV, and UC fractions (Figure 10). The panel reflects the major functional groups of the human erythrocyte membrane proteome, including immune evasion-related proteins CD47, CD55, and CD59, cytoskeletal proteins SPTA1, SPTB, ANK1, EPB41, EPB42, ACTG1, and DMTN, transmembrane proteins SLC4A1 and AQP1, lipid raft-associated proteins (FLOT1, FLOT2, and STOM), and the glycoprotein glycophorin-A (GYPA). Values close to zero indicate similar abundances between fresh and frozen preparations, positive values indicate higher abundances in frozen preparations, and negative values indicate lower abundances in frozen preparations.

**Figure 10:**
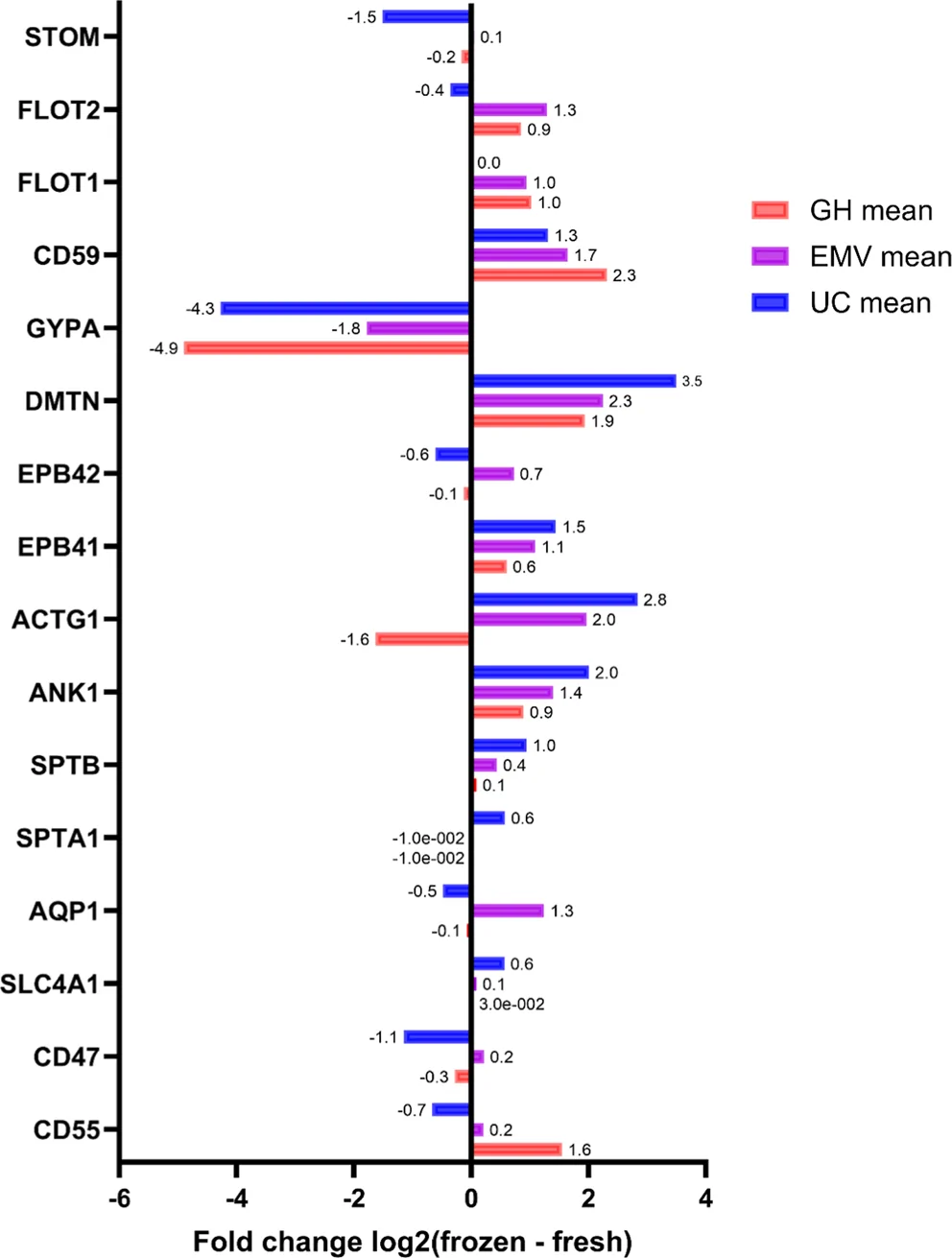
Log2 fold change of selected erythrocyte membrane proteins in frozen relative to fresh preparations across GH, EMV, and UC fractions. Positive values indicate higher abundance in frozen preparations; negative values indicate higher abundance in fresh preparations. Abbreviations are defined in the main text.

Band 3 (SLC4A1) showed a minimal change in GH (0.03) and EMV (0.1) fractions, with a slightly positive value in UC (0.6). Spectrin alpha (SPTA1) showed near-zero values in GH (−0.01) and EMV (−0.01), with a modest positive value in UC (0.6). Spectrin beta (SPTB) showed similarly stable profiles in GH (0.1) and EMV (0.4), with a modest positive value in UC (1.0). Ankyrin-1 (ANK1) showed increasing values across fractions (GH, 0.9, EMV, 1.4, UC, 2.0). Dematin (DMTN) showed the largest positive fold change among cytoskeletal proteins, particularly in UC (UC, 3.5, GH, 1.9, and EMV, 2.3). These results indicate that the core membrane proteome, encompassing key structural and functional membrane proteins, is largely preserved in GH and EMV fractions following a single freeze-thaw event. Conversely, UC fractions showed a more pronounced enrichment in frozen preparations for several cytoskeletal proteins. Band 3 (SLC4A1) remained stable across all fractions, which is particularly relevant, given its dual role as the primary anion exchanger and the central scaffold for cytoskeletal attachment in erythrocytes.

Among immune evasion markers, CD55 showed a positive value in GH (1.6) but near-zero or slightly negative values in EMV (0.2) and UC (−0.7). CD59 showed consistently positive values across all fractions (GH: 2.3, EMV: 1.7, UC: 1.3). Lastly, CD47 showed a slight decrease in frozen preparations, most pronounced in UC (−1.1), with values of −0.3 in GH and 0.2 in EMV. CD47 is a transmembrane protein that inhibits phagocytic clearance by binding SIRPα on macrophages and dendritic cells, and its retention on EMVs is considered essential for the immune evasion properties of RBC-derived vesicles.^11^ These differences may be critical for applications where preservation of specific surface proteins is required, such as immune evasion or targeted drug delivery. Whether the reduction observed in UC fractions translates to functionally relevant differences in macrophage evasion would require direct in vitro phagocytosis assays, which represent a logical next step for validation of frozen blood as an EMV source.

The most notable change was observed for GYPA, which showed the largest negative fold changes across all fractions (GH: −4.9, EMV: −1.8, and UC: −4.3), indicating substantially lower abundance in frozen compared to fresh preparations. GYPA is the major sialoglycoprotein of the erythrocyte outer leaflet and one of the primary carriers of sialic acid residues that generate the negative surface charge of the RBC membrane. The selective reduction of GYPA in frozen preparations is consistent with known RBC membrane protein sorting during vesiculation: Band 3 and GYPA are selectively depleted from released vesicles relative to freely mobile lipid-anchored proteins, with the degree of depletion reflecting the strength of cytoskeletal anchoring.^44^ A single freeze-thaw event may alter the binding affinity of GYPA to the cytoskeleton and thereby change the efficiency of its incorporation into ghost membrane preparations during hypotonic lysis.

Actin (ACTG1) showed a divergent pattern, a negative value in GH (−1.6), but positive values in EMV (2.0) and UC (2.8). Stomatin (STOM) showed near-zero values in GH (0.1) and EMV (−0.2) but a negative value in UC (−1.5). Changes in cytoskeletal protein composition, specifically increased membrane association of actin, have been reported in RBC membranes following mechanical and oxidative stress,^41^ conditions imposed on erythrocytes during a single freeze-thaw event without cryoprotectant. The elevated level of ACTG1 in frozen EMVs may reflect reorganization of the spectrin-actin network, with partial redistribution of actin to the membrane fraction.

Nevertheless, determining the functional relevance of these proteomic differences will require complementary biological studies, including in vivo assessment of parameters such as circulation time, biodistribution, tissue accumulation, and clearance, as well as functional assays to establish their biological consequences. Thus, while the observed differences provide biologically informative insights, their functional significance cannot be conclusively established from proteomic data alone without further biological validation.

## 4 Conclusions

This study demonstrates that EMVs can be successfully generated from both fresh and frozen blood, with key physicochemical and structural properties largely preserved despite differences in blood storage conditions. Although freezing introduces heterogeneity and membrane damage at the erythrocyte ghost stage, the extrusion process effectively normalizes particle size, yielding vesicles with comparable hydrodynamic diameters and size distributions regardless of the blood storage condition. Subsequent ultracentrifugation further refines vesicle populations, producing more homogeneous subfractions and partially mitigating the morphological irregularities introduced by freezing.

Across all preparation stages, EMVs retained a stable negative surface charge within physiologically relevant ranges, indicating a preserved colloidal stability and membrane composition. Complementary sizing techniques highlighted the importance of methodological differences, with nanoflow cytometry revealing smaller, more abundant vesicle populations that are not captured by bulk methods such as DLS. Structural analyses confirmed that both fresh and frozen EMVs form intact, bilayer-enclosed vesicles, with cryo-TEM validating their native spherical morphology and identifying freezing-associated membrane disruptions that are largely resolved after purification.

Proteomic analysis revealed that while the overall membrane protein composition is largely conserved following a freeze–thaw cycle, freezing reduces proteomic complexity in membrane fractions and selectively depletes cytosol-associated proteins. Importantly, the core erythrocyte membrane proteome, including key structural proteins such as Band 3, remains stable, supporting the integrity of vesicle membranes derived from frozen blood. However, specific alterations, such as reduced glycophorin A and CD47 expression levels and changes in cytoskeletal protein association, suggest that freezing may influence functional properties of EMVs, particularly those related to immune interactions and membrane organization.

Overall, our findings indicate that frozen blood represents a viable and practical source for EMV production, especially when the immediate processing of fresh samples is not feasible. While minor structural and proteomic differences exist, they do not substantially compromise vesicle formation or stability. Nevertheless, potential functional consequences of altered protein composition should be further investigated, particularly in applications where biological activity, targeting, or immune evasion is critical.

## Supplementary Material





## Data Availability

The proteomic dataset supporting the conclusions of this article is available in the ProteomeXchange Consortium via the PRIDE partner repository with the dataset identifier PXD078491.

## Notes

SDS-PAGE gel images showing protein expression profiles in WE, GH, EMV, and UC fractions from fresh and frozen blood preparations And protein matrix with log2-transformed label-free quantification intensities included in the final filtered dataset for each sample type (WE, GH, EMV, and UC fractions from fresh and frozen blood preparations) (PDF)
Tomic_et_al_JPR_2026_TableS1.xlsx (XLSX)

## Abbreviations

ANK1: ankyrin-1
ACTG1: actin gamma 1
AQP1: aquaporin-1
BCA: bicinchoninic acid
BSA: bovine serum albumin
CPD-SAGM: citrate phosphate dextrose–saline adenine glucose mannitol
cryo-TEM: cryogenic transmission electron microscopy
DCC: density-changing centrifugation
DLS: dynamic light scattering
DMTN: dematin
DNA: deoxyribonucleic acid
EC: extracellular
EDTA: ethylenediaminetetraacetic acid
EMV: erythrocyte membrane vesicle
EPB41: erythrocyte membrane protein band 4.1
EPB42: erythrocyte membrane protein band 4.2
EV: extracellular vesicle
FLOT1: flotillin-1
FLOT2: flotillin-2
GH: ghost
GO: gene ontology
GYPA: glycophorin-A
HCD: higher-energy collisional dissociation
HPLC: high-performance liquid chromatography
IQR: interquartile range
MISEV: minimal information for studies of extracellular vesicles
MS: mass spectrometry
NS-TEM: negative-staining transmission electron microscopy
NTA: nanoparticle tracking analysis
PAGE: polyacrylamide gel electrophoresis
PANTHER: protein analysis through evolutionary relationships
PBS: phosphate-buffered saline
PDI: polydispersity index
PEG: polyethylene glycol
PRIDE: Proteomics Identifications Database
QC: quality control
RBC: red blood cell
RNA: ribonucleic acid
SD: standard deviation
SDS: sodium dodecyl sulfate
SIRPα: signal regulatory protein alpha
SLC4A1: solute carrier family 4 member 1 (Band 3)
SPTA1: spectrin alpha 1
SPTB: spectrin beta
SSC: side scatter
STOM: stomatin
TC: trisodium citrate
TEM: transmission electron microscopy
UC: ultracentrifuge fraction
WE: whole erythrocytes
