# Supplementary material for: Proteomic and Physicochemical Analysis of Erythrocyte Membrane Vesicles from Fresh and Frozen Blood
Source: J Proteome Res. 2026 Sep 9;25(10):5534–48. doi: 10.1021/acs.jproteome.6c00535 (PMC13640506; doi:10.1021/acs.jproteome.6c00535)
Supplement: Supplementary file 1 [file pr6c00535_si_001.pdf]

SUPPORTING INFORMATION FILE

Proteomic and physicochemical analysis of erythrocyte membrane vesicles from fresh and frozen blood

Katarina Tomić [1,2§], Sara Ivanovski [2,3§], Jernej Šribar [4], Daša Čebulj [1,5], Samuel Žvanut [6], Metka Lenassi [6], Marko Fonović [3], Igor Križaj [4], Nina Kostevšek [1,2]*

[1] Department of Nanostructured Materials, K7, Jožef Stefan Institute, Ljubljana 1000, Slovenia

[2] Jožef Stefan International Postgraduate School, Jožef Stefan Institute, Ljubljana 1000, Slovenia

[3] Department of Biochemistry and Molecular and Structural Biology, B1, Jožef Stefan Institute, Ljubljana 1000, Slovenia

[4] Department of Molecular and Biomedical Sciences, B2, Jožef Stefan Institute, Ljubljana 1000, Slovenia

[5] University of Ljubljana, Faculty of Chemistry and Chemical Technology, Ljubljana 1000, Slovenia

[6] Institute of Biochemistry and Molecular Genetics, Faculty of Medicine, University of Ljubljana, Ljubljana 1000, Slovenia

*E-mail: katarina.tomic@ijs.si

§K.T. and S.I. contributed equally to this work

Table of contents:

Supplemental figure S1: SDS-PAGE gel images showing protein expression profiles in WE, GH, EMV, and UC fractions from fresh and frozen blood preparations.

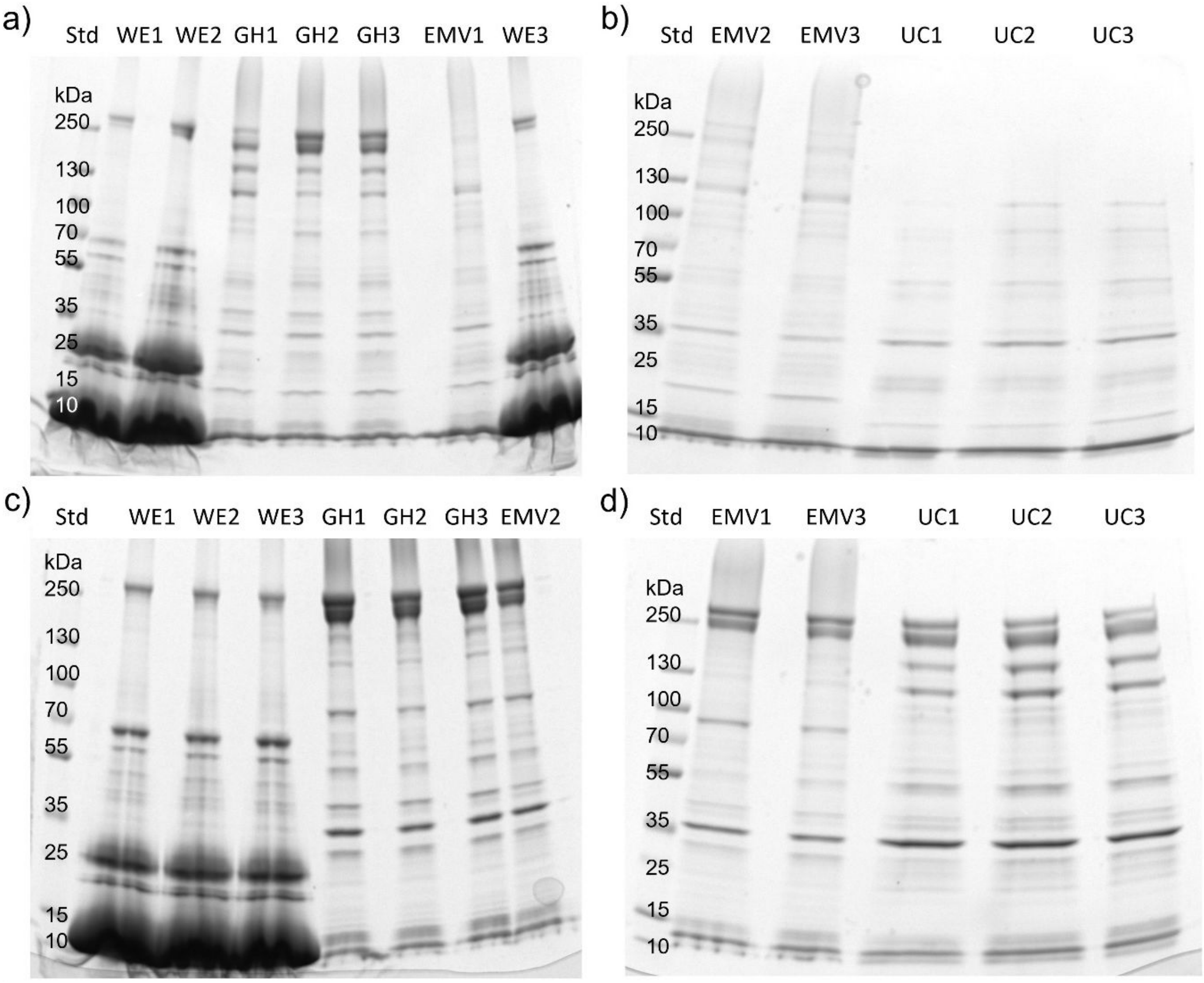

Figure S1. SDS-PAGE analysis of protein expression in whole erythrocytes (WE), erythrocyte ghosts (GH), erythrocyte membrane vesicles (EMVs), and ultracentrifuged samples (UC), for both groups of samples, i.e., prepared from a) and b) fresh or c) and d) frozen blood. Each sample was loaded into the gel in triplicate with a protein concentration of 14 µg for

electrophoretic running. Proteins were visualized by Coomassie Brilliant Blue staining. STD: standard protein ladder
